# Biomimetic Nucleic Acid Drug Delivery Systems for Relieving Tumor Immunosuppressive Microenvironment

**DOI:** 10.3390/pharmaceutics16081028

**Published:** 2024-08-01

**Authors:** Wenlu Yan, Ying Cao, Qi Yin, Yaping Li

**Affiliations:** 1 State Key Laboratory of Drug Research and Center of Pharmaceutics, Shanghai Institute of Materia Medica, Chinese Academy of Sciences, Shanghai 201203, China; s20-yanwenlu@simm.ac.cn (W.Y.); caoyiing@163.com (Y.C.); 2 University of Chinese Academy of Sciences, Beijing 100049, China; 3 School of Life Sciences, Jilin University, Changchun 130012, China; 4 Yantai Key Laboratory of Nanomedicine and Advanced Preparations, Yantai Institute of Materia Medica, Yantai 264000, China; 5 Shandong Laboratory of Yantai Drug Discovery, Bohai Rim Advanced Research Institute for Drug Discovery, Yantai 264000, China

**Keywords:** biomimetic, drug delivery system, nucleic acid, immunotherapy, tumor immunosuppressive microenvironment

## Abstract

Immunotherapy combats tumors by enhancing the body’s immune surveillance and clearance of tumor cells. Various nucleic acid drugs can be used in immunotherapy, such as DNA expressing cytokines, mRNA tumor vaccines, small interfering RNAs (siRNA) knocking down immunosuppressive molecules, and oligonucleotides that can be used as immune adjuvants. Nucleic acid drugs, which are prone to nuclease degradation in the circulation and find it difficult to enter the target cells, typically necessitate developing appropriate vectors for effective in vivo delivery. Biomimetic drug delivery systems, derived from viruses, bacteria, and cells, can protect the cargos from degradation and clearance, and deliver them to the target cells to ensure safety. Moreover, they can activate the immune system through their endogenous activities and active components, thereby improving the efficacy of antitumor immunotherapeutic nucleic acid drugs. In this review, biomimetic nucleic acid delivery systems for relieving a tumor immunosuppressive microenvironment are introduced. Their immune activation mechanisms, including upregulating the proinflammatory cytokines, serving as tumor vaccines, inhibiting immune checkpoints, and modulating intratumoral immune cells, are elaborated. The advantages and disadvantages, as well as possible directions for their clinical translation, are summarized at last.

## 1. Introduction

Immunotherapy, as an alternative cancer treatment following surgery, chemotherapy, and radiotherapy, has been applied in clinical practice by regulating the body’s immune system to kill tumors [1,2]. The interaction between the immune system and tumor involves the recognition and killing of tumors by the immune system, as well as evading immunosurveillance by tumors [3,4]. Based on this, the strategies of immunotherapy primarily focus on enhancing antitumor immune responses such as cytokines, vaccines, adjuvants, and adoptive cell transfer therapy, or reversing immune suppression such as immune checkpoint blockade and elimination of immunosuppressive cells [5]. Numerous immunotherapeutic agents have been approved by the Food and Drug Administration (FDA) due to their favorable clinical responses [6,7]. Nevertheless, immunotherapy is still in its infancy as only a small proportion of patients benefit from it, highlighting the urgent need for the development of more effective and safer immunotherapeutic strategies [8,9].

Nucleic acid drugs, with the advantages of possessing abundant and clear therapeutic targets, design simplicity, and long-lasting efficacy, can be majorly categorized into the following groups: microRNA (miRNA), small interfering RNA (siRNA), and antisense oligonucleotide (ASO) for gene silence by degrading target RNA; plasmid DNA and mRNA for introducing target genes; and the clustered regularly interspaced short palindromic repeats (CRISPR)/Cas system for versatile regulation of genes [10,11,12,13,14]. Nucleic acid drugs have been widely used in antitumor immunotherapy [15]. For example, siRNA targeting immune checkpoints can alleviate immunosuppression, and it can inhibit the expression of target proteins more efficiently and specifically compared with antibodies or small molecular drugs [16,17]; mRNA tumor vaccines can trigger systematic immune responses, and they can be designed to produce various antigen proteins efficiently with the bioreactors in the cells of vaccinated patients [18,19]; and the CRISPR/Cas system can provide convenient and accurate tools for manipulating genes such as designing multifunctional chimeric antigen receptor (CAR)-T cells and regulating undruggable targets [20,21]. Furthermore, nucleic acids from viruses or bacteria can serve as effective adjuvants due to their strong immunostimulant ability [22,23,24]. Nucleic acid drugs have to overcome a series of biological barriers to enter target cells after administration, which necessitates the discovery of strategies to improve their stability, circulation time, cellular uptake, and endosomal escape [25,26]. Chemical modifications including sugar, backbone, nucleobase, and 3′- and 5′-terminal modifications have been developed to enhance the stability, affinity, and delivery efficiency of nucleic acids [27]. For instance, phosphorothioate backbone can improve oligonucleotide stability; cholesterol, alkyl chain, or vitamin E modification can increase the lipophilicity and cellular uptake of therapeutic nucleic acids; *N*-acetylgalactosamine (GalNAc) conjugation can improve the hepatic delivery of oligonucleotides [28]. Delivery systems including polymer micelles, liposomes, and lipid nanoparticles can improve the in vivo behaviors of nucleic acids by encapsulating the cargos to protect them against nucleases, penetrating the biological barriers to reach the lesion specifically, preventing their non-specific binding to proteins and realizing endosomal escape [29,30]. Despite the fact that several mRNA-loaded lipid nanoparticle vaccines are undergoing clinical trials and yielding preliminary benefits [31], there is still a need to develop novel nucleic acid delivery systems to fulfill additional needs such as extrahepatic delivery and enhanced biosafety.

In addition to the delivery efficiency, the compensation of a tumor immunosuppressive microenvironment (TIME) hampers the outcomes of immunotherapeutic nucleic acid agents [32,33,34]. Tumor immunosuppression results from multiple levels: (i) evading recognition by immune cells due to low mutational burden and immunogenicity [35,36]; (ii) escaping the attack from immune cells by upregulating immune checkpoints [37]; (iii) inhibiting the infiltration of immune cells by dense extracellular matrix and chemokines [38]; (iv) immunosuppressive cells and cytokines in the tumor microenvironment [39,40]; and (v) intratumoral physicochemical properties such as weak acidity and hypoxia, and abnormal metabolic status [41,42,43]. Regarding the complexity of TIME, it is prospective to combine nucleic acid drugs with other therapeutic strategies to synergistically activate antitumor immunity.

**Figure 1:**
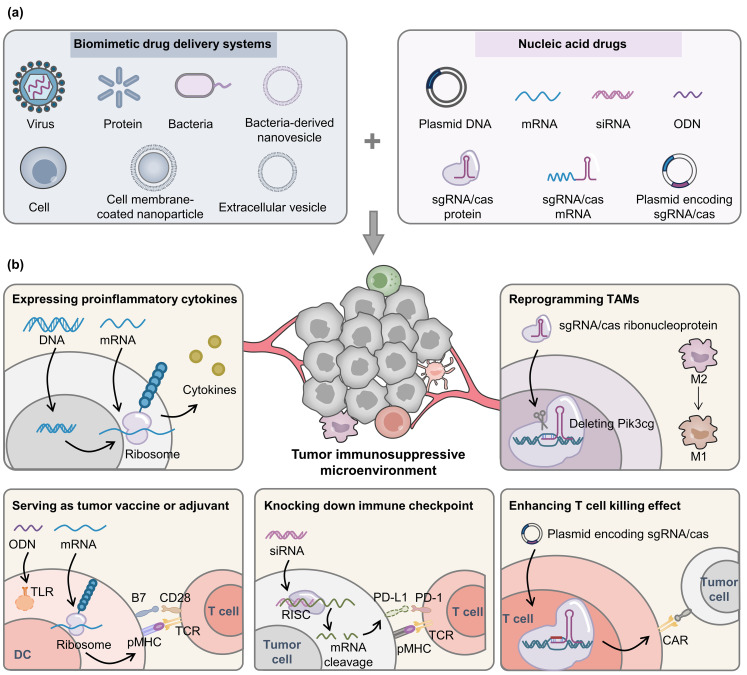
Biomimetic nucleic acid drug delivery systems for relieving tumor immunosuppressive microenvironment. (**a**) Biomimetic platforms derived from viruses/bacteria/cells serve as nucleic acid drug delivery systems. (**b**) The mechanisms of relieving tumor immunosuppressive microenvironment, including expressing proinflammatory cytokines, serving as vaccines or adjuvants, knocking down immune checkpoint molecules, reprogramming tumor-associated macrophages, and enhancing the killing effect of T cells. ODN, oligonucleotides; TLR, Toll-like receptor; DC, dendritic cell; pMHC, peptide-major histocompatibility complex; TCR, T cell receptor; RISC, RNA-induced silencing complex; PD-1/PD-L1, programmed cell death 1/PD ligand 1; TAM, tumor associated macrophage; CAR, chimeric antigen receptor.

Biomimetic drug delivery systems, derived from cells, bacteria, and viruses, can improve the delivery efficiency with their targeting ability and long circulation time, and activate immune systems with their endogenous activities and active components [44,45,46], thus improving the safety and therapy effects. In this review, biomimetic nucleic acid delivery systems for antitumor immunotherapy are introduced (Figure 1). Their design strategies and mechanisms of regulating TIME are highlighted. Finally, the current progress and future prospects of this field are discussed.

## 2. Biomimetic Nucleic Acid Delivery System for Antitumor Immunotherapy

### 2.1. Virus-Derived Delivery Systems

The viral structure, consisting of a protein shell and a nucleic acid core, confers its potential as a natural nucleic acid carrier [47]. Viruses containing genes of interest can infect target cells and achieve nucleic acid delivery. Besides complete viruses, multiple proteins with the ability to bind and encapsulate nucleic acid to form virus-like particles are also promising vectors [48,49].

#### 2.1.1. Virus

Viral vectors, one kind of the earliest tools for nucleic acid drug delivery, can deliver DNA or mRNA sequences that encode therapeutic agents, CRISPR/Cas systems, and oligonucleotides [50]. Lentiviruses, retroviruses, adenoviruses, and adeno-associated viruses (AAVs) are commonly used vectors. Lentiviruses and retroviruses infect cells and integrate the target genes into the genome of host cells, which may lead to genotoxicity [51,52]. Therefore, these two viruses are not suitable for in vivo therapy but are used for in vitro gene editing of cells, such as the preparation of CAR-T cells [53]. Adenoviruses, with high gene transduction efficiency, replicate in the host cell nucleus to accomplish transient target gene expression without integration, which is suitable to generate short-term and high-level therapeutic benefits [54]. AAVs, requiring the help of a helper virus to complete replication, can persist in host cells for a long time and are suitable for therapies that require long-term and stable gene expression, such as gene therapy, for inherited diseases [55]. The aforementioned viruses have been utilized in the treatment of a wide range of refractory diseases, and in particular, adenoviruses and AAVs have assisted in antitumor gene therapy for a long time [56].

**Figure 2:**
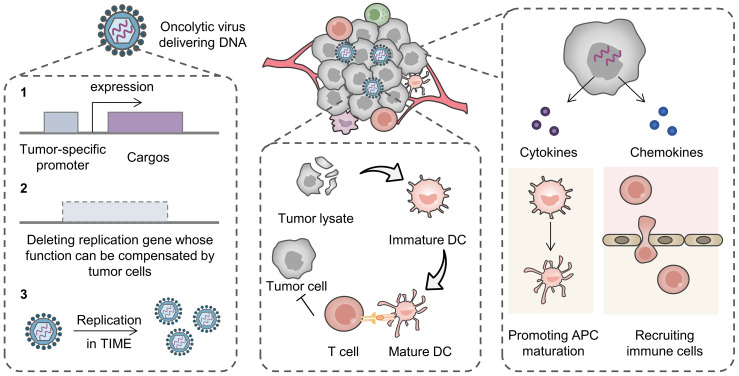
Oncolytic virus-based nucleic acid drug systems for activating antitumor immune responses. The tumor specificity of an oncolytic virus can be improved by introducing tumor-specific promoters to control the expression of therapeutics (1) and deleting replication genes whose function can be compensated by tumor cells (2). TIME is also conducive for virus replication (3). Tumor lysate after treatment with an oncolytic virus serves as a tumor vaccine, which can maturate DC and activate tumor-killing effects of T cells. In addition, therapeutics encoded by the genes carried by the virus, such as cytokines and chemokines, can promote APC maturation and recruiting immune cells, respectively. TIME, tumor immunosuppressive microenvironment; DC, dendritic cell; APC, antigen-presenting cell.

Delivery of nucleic acid drugs that express proinflammatory cytokines enhances antitumor immunity. Proinflammatory cytokines need to be fast-acting or they may promote tumor progression through chronic inflammatory pathways [57,58], and thus, adenoviruses are potential vectors. Interferons (IFNs) can directly induce apoptosis of tumor cells and enhance antitumor immunity by increasing the activity of various immune cells such as dendritic cells (DCs), cytotoxic T cells, and proinflammatory macrophages [59]. Adenoviral vectors delivering genes encoding IFN-α or IFN-β have yielded clinical benefits by augmenting antitumor immune responses [60,61]. ONCOS-102, an oncolytic virus in clinical use, was constructed by introducing a granulocyte macrophage colony stimulating factor (GM-CSF) gene into a tumor-selective replicative oncolytic adenovirus [62,63,64]. The oncolytic adenovirus specifically lysed tumors and released tumor antigens; at the same time, GM-CSF induced antigen-presenting cell (APC) maturation and natural killer (NK) cell infiltration, exerting synergistic antitumor effects. In addition, a range of adenoviruses have been developed to deliver genes encoding CD40 ligands to promote B cell and APC activation [65], interleukin (IL)-12 to enhance cytotoxic T cell and NK cell-killing effects [66], and chemoattractant cytokine ligand (CCL)-5 chemokines to recruit antitumor immune cells [67] (Figure 2).

Tumor vaccines stimulate antitumor immunity, and viruses amplify immune stimulation due to their immunogenicity. Therefore, viruses delivering genes encoding tumor antigens present an effective platform, in which viruses act as a vector as well as an active component. An attenuated strain of vaccinia virus expressing both a tumor-associated antigen and IL-12 was designed to induce antitumor responses [68]. The multi-component vaccine prolonged the survival time of patients when combined with first-line chemotherapy, providing a viable adjuvant tumor therapy strategy.

The challenge of uncontrolled replication of virus-based therapeutics in vivo needs to be addressed. To achieve the dual goals of rapid virus replication for in vitro preparation and non-replication for in vivo safety, premature termination codons (PTC) encoding proteins for viral replication in specific systems in vitro and acting as a termination codon in vivo can be introduced into the virus [69]. The engineered virus maintaining both infectivity and immune activation effects is an intelligent drug delivery platform. CpG oligonucleotides are synthetic agonists of Toll-like receptor (TLR) 9, which can activate cellular and humoral immunity against specific antigens. Ji et al. constructed a vaccine adjuvant-integrated influenza A viral delivery system by anchoring CpG and antigen peptide on the surface of the virus [70]. The platform achieved lung targeting with the natural lung tropism of influenza virus and initiated antitumor immune responses, significantly inhibiting lung metastasis from melanoma, colorectal, and breast cancers.

Although some viral drug delivery systems have been approved by the FDA for clinical use, virus-based nucleic acid drug delivery still faces many challenges, including complex preparation processes, expensive costs, rapid clearance, restricted size of target gene fragments, and biosafety issues [71]. Notably, various strategies have been developed for improving the biocompatibility of the viral drugs with the advances of biotechnology. For example, knocking out disease-causing genes or modulating viral replication ability enables many notorious viruses as vectors [72]. Tumor-specific promoters can be introduced into the gene sequence to achieve tumor site-specific expression of the delivered nucleic acid [73]. Tumor site-specific viral duplication can also be achieved by deleting genes critical to viral replication whose function can be compensated by tumor cells [74]. In addition, tumors with immunosuppression are more susceptible to virus infection [75] (Figure 2).

#### 2.1.2. Virus-like Particles

The structural characteristics of viruses have inspired the development of nucleic acid delivery systems. Virus-like particles have been devised in virtue of the natural affinity and packaging capabilities of proteins for nucleic acids, shielding nucleic acids from enzymatic degradation while facilitating their uptake by cells [48,76,77]. The main strategies for constructing protein-based nucleic acid delivery systems include preparing protein nanocages to encapsulate nucleic acids, and using positively charged proteins to compress nucleic acids by electrostatic interactions.

Ferritin, as an endogenous protein with excellent biocompatibility, can self-assemble to form a hollow nanocage-like structure to encapsulate cargos. To enhance nucleic acid loading of human heavy-chain ferritin (HFn), acidic amino acids in the protein cavity were replaced with basic amino acids. Bioengineered HFn was efficiently loaded with the TLR agonist CpG or miRNA29a and delivered them to DCs, inducing systemic antitumor immune responses when synergized with photodynamic therapy [78]. Virus-derived coat proteins can also encapsulate nucleic acids. Encapsulation of prostatic acid phosphatase and GM-CSF-expressing mRNAs with phage capsid proteins formed phage-like particles that stimulated humoral and cellular immune responses against prostate cancer in mice [79].

Cationic proteins such as protamine can bind tightly to nucleic acids to form protein–nucleic acid complexes. Meng et al. constructed a virus-like vaccine particle with a protamine–mRNA core and a phospholipid bilayer shell. The immune adjuvant CpG was adsorbed in the core as well. Virus-mimicking mRNA vaccines stimulated antigen presentation by DCs, promoted intratumoral infiltration of T cells, and inhibited tumor growth [80]. Similarly, a cationic liposome–protamine complex mRNA vaccine achieved strong intranasal immunization and suppressed an aggressive Lewis lung cancer [81].

Natural nucleic acid delivery systems existing in the organism can mediate intracellular RNA delivery. In mammals, there exist many retrotransposon-associated proteins which can bind to RNA. Through a systematic search for retrotransposon proteins in the human genome, Segel et al. identified PEG10 as an efficient RNA carrier protein [82]. Selective endogenous encapsidation (SEND) for cellular delivery was developed by selectively encapsulating RNA consisting of both the 5′ and 3′ untranslated region (UTR) with PEG10. SEND could serve as an all-in-one vector for the CRISPR/Cas system by co-packaging sgRNA (single guide RNA) and mRNA encoding Cas9. Human paraneoplastic antigen Ma2 is also capable of forming virus-like capsids for nucleic acid drug delivery [83]. Endogenous proteins are safer than viruses and cationic liposomes as nucleic acid drug delivery systems. However, its encapsulation rate for different kinds of nucleic acid drugs still needs to be further explored and optimized.

### 2.2. Bacteria-Derived Delivery Systems

**Figure 3:**
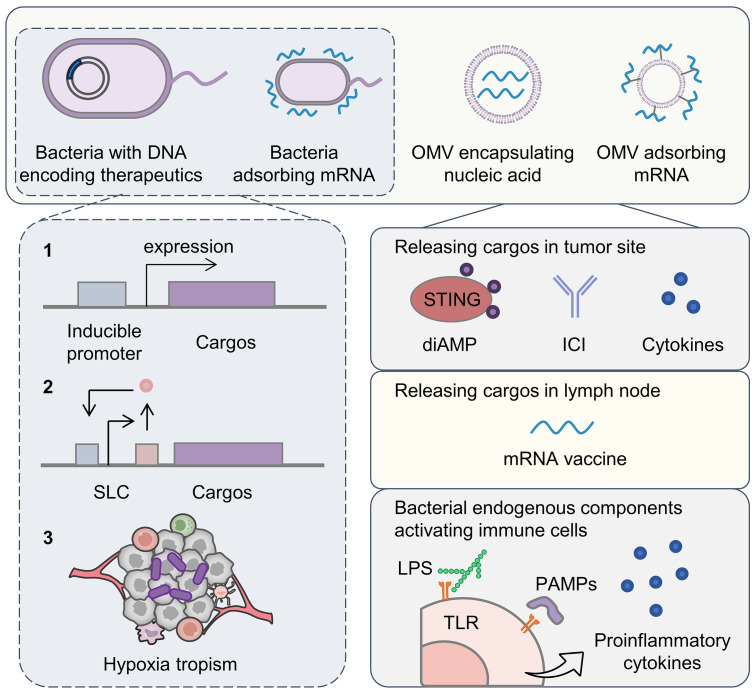
Bacteria-derived nucleic acid drug delivery systems for antitumor immunotherapy. Bacteria and outer membrane vesicles (OMVs) can both encapsulate and adsorb the nucleic acid drugs. The controllability of bacteria can be improved by introducing an inducible promoter (1) or synchronized lysis circuit (SLC) (2) into the gene circuits. Additionally, anaerobic bacteria can target a tumor with their hypoxia tropism (3). Bacteria-derived nucleic acid drug delivery systems release cargos in the tumor site or lymph node, whose effects can be further enhanced by the bacterial endogenous components with immune activation ability. STING, stimulator of interferon gene; ICI, immune checkpoint inhibitor; LPS, lipopolysaccharides; TLR, Toll-like receptor; PAMPs, pathogen-associated molecular patterns.

Bacteria have been recognized as a potential tool for the treatment of tumors since the 19th century [84]. Bacteria can be used both to treat diseases directly, such as probiotics, and as carriers for drug delivery [85]. Larger-sized bacteria can carry more cargo, such as DNA, mRNA, or nanoparticles, than viruses can. Extracellular vesicles from bacteria have also been widely used in drug delivery due to their rich active contents and in vivo behavior [86] (Figure 3).

#### 2.2.1. Bacteria

Bacterial surfaces have abundant motifs that can be modified to carry nucleic acid drugs. Target genes can also be introduced into the bacterial genome and can be translated upon reaching specific sites [87]. Bacteria as nucleic acid delivery systems for immunotherapy possess the following advantages: (i) immunostimulatory activity relieving TIME; (ii) tendency to colonize hypoxic tumors (anaerobic bacteria) enabling tumor targeting; and (iii) suitability for modifying genomes. The safety of bacteria can be improved by deleting pathogen-associated genes [88].

Facultative anaerobes photosynthetic bacteria (PSB) with photosensitivity and hypoxia-targeting ability provide a kind of multifunctional platform for antitumor drug delivery. Zhang et al. adsorbed nanosheets incorporating a tumor mRNA vaccine onto PSB, constructing a system for the combination of immunotherapy and photothermal therapy [89]. The PSB accumulated at tumor sites after administration and released the mRNA vaccine after photothermal conversion. Photothermal therapy induced the immunogenic cell death (ICD) of the tumor to release damage-associated molecular patterns (DAMPs), which promoted the maturation of DCs when combined with the mRNA vaccine. The PSB-based mRNA vaccine delivery system inhibited tumor progression by activating antitumor immune responses and immune memory, demonstrating its promising nucleic acid delivery ability.

Genes expressing therapeutic proteins can be introduced into the bacteria with a complete protein synthesis system to form a drug production factory. Bacteria engineered via synthetic biology have been constructed to deliver genes specifically to tumor sites and release therapeutics in a controllable manner [90]. For example, a hypoxia-inducible promoter was introduced into the gene circuit in the *Escherichia coli* (*E. coli*) Nissle to control the expression of stimulator of interferon genes (STING) agonists [91]. STING agonist cyclic diAMP was only released in hypoxic tumor sites for activating APCs. The stimulation of the STING pathway and pattern recognition receptor amplified antitumor immunity and combatted the tumor effectively in a clinical trial [92]. Biosensors detecting oxygen levels, lactate levels, and pH levels can also be introduced to modulate the tropism of the bacteria [93]. Synchronized lysis circuits (SLC) that can sense quorum density to control the expression and release of proteins corresponding to carried genes can be introduced into bacterial vectors to achieve the controlled release of therapeutics [94]. The interaction of CD47 and signal regulatory protein (SIRP) α provides a “do not eat me” signal to inhibit tumor phagocytosis by macrophages, and thus, antibodies against CD47 or SIRP have been developed for reserving immunosuppression [95]. An *E. Coli* containing a gene expressing a CD47 antagonist and an SLC was constructed for a locally released CD47 antagonist in tumors [96]. Similarly, the genes encoding immune checkpoint inhibitors or proinflammatory chemokines were also introduced in the bacteria with SLC to prevent dose-related drug toxicity [97].

#### 2.2.2. Bacteria-Derived Nanovesicle

Outer membrane vesicles (OMVs) derived from Gram-negative bacteria show potential for nucleic acid drug delivery because of their intrinsic nano-size and intercellular interactions [98]. 

OMVs inherit components from bacteria with abundant pathogen-associated molecular patterns (PAMPs), which enable the efficient uptake and stimulation of DCs. To utilize the effective DC uptake of and activation by OMVs, Li et al. displayed mRNA on the surface of OMVs via molecular glue linking [99]. mRNA was processed and presented by DCs with the assistance of OMVs to enhance the tumor-killing ability of CD8^+^ T cells, resulting in a complete tumor regression in 37.5% of colorectal cancer mouse models. The “Plug-and-Display” strategy was simpler and faster than the conventional encapsulation strategy, and more suitable for personalized mRNA vaccine delivery. When designing OMV as a vector, its immunogenicity resulting in rapid clearance needs to be addressed. Tumor microenvironment-sensitive linker-conjugated albumin or biomimetic mineralization on OMVs reduced side effects and mononuclear phagocyte system clearance [100,101].

Bacterial cytoplasmic membrane vesicles containing cytosolic proteins, DNA, RNA, and secreted proteins can also serve as nucleic acid vectors. The anti-inflammatory M2-phenotype tumor-associated macrophages (TAMs) promote tumor growth and metastasis. Reprogramming TAMs toward the M1 type can alleviate immunosuppression [102]. However, reprogrammed macrophages are phenotypically unstable under the influence of immunosuppressive cytokines in a tumor microenvironment. Knockout of M2-type TAM-related genes by gene engineering is expected to result in permanent regulation of TAMs. In the study conducted by Zhao et al., a Cas9-sgRNA ribonucleoprotein targeting a TAM reprogramming switch Pik3cg and a CpG-rich DNA fragment as a potent TLR9 ligand were loaded into the bacterial cytoplasmic membrane vesicles using plasmid-transformed *E. coli* as a production platform [103]. The nanovesicles were further decorated with pH-responsive polyethylene glycol (PEG) to prolong circulation time and galactosamine to deliver cargos targeting macrophages. Through stable reprogramming of the TAM phenotype, the nanovesicle transformed the tumor microenvironment from a “cold” state to a “hot” one with an increase in antitumor immune cells and cytokines. This in vivo CRISPR/Cas9 platform, based on bacterial cytoplasmic vesicles with a simple fabrication process and precise delivery, paves the path for gene-modulating immunotherapy.

### 2.3. Cell-Derived Delivery Systems

Cells and their derivatives, including cell membrane and extracellular vesicles, can be used as drug carriers to enhance the therapeutic efficacy by improving the pharmacokinetic properties of drugs with their long circulation ability, increasing drug accumulation in target tissues with their tissue-homing ability, promoting drug penetration to cross biological barriers, and exerting their endogenous activities [104].

#### 2.3.1. Cells

A variety of cells have been developed as drug delivery systems due to their unique biological properties, such as erythrocytes with a long lifespan and long-circulating capacity, neutrophils and macrophages targeting inflammatory sites, and NK cells and T cells that can exert tumor-killing effects [105]. Utilizing cells as drug delivery vehicles involves several primary processes: cell collection (from in vivo sources or cell lines), purification and identification, cell expansion, cell modification, drug loading, quality assessment, and reinfusion [106]. Drug loading can be achieved either by surface modification or by endocytosis. Considering the instability of nucleic acid drugs, which may be inactivated by intracellular enzymes after endocytosis, surface coupling is more suitable for the loading of nucleic acid drugs.

Tumor cells are the most complete providers of tumor antigens to elicit individualized vaccination effects [107]. Lysed tumor cells have been used to treat various cancers in clinical trials, which compensates for the low responsiveness of a single-antigen vaccine [108]. Furthermore, adhesion molecules expressed on the cells can mediate targeting capabilities [109]. To eliminate the carcinogenicity, repeated freezing–thawing has been carried out to obtain tumor cell corpses as a versatile drug vector. Cryo-shocked tumor cells have been utilized to deliver chemotherapeutic agents, immune checkpoint inhibitors, liposomes, and CRISPR/Cas systems [110,111,112]. Cryogenic silicification, which preserves the cell integrity and protein function as well as facilitates surface modification, provides a simple method for a whole tumor cell-based vaccine. Guo et al. first adsorbed positively charged polyethyleneimine (PEI) on the surface of cryo-silicified tumor cells, which was negatively charged due to silanol groups, and subsequently coated the cells with pathogen-mimicking coatings (CpG and monophosphoryl lipid A) to form a cell-based vaccine and adjuvant delivery platform [113]. The TLR agonists CpG and monophosphoryl lipid A enhanced the vaccine uptake of DCs by 9 times and activated them. The silicified tumor cells acted both as a prophylactic vaccine to prevent tumorigenesis and as a therapeutic vaccine to prolong the survival of tumor-bearing mice by promoting function of tumor-associated effector lymphocytes. In conclusion, the silicified tumor cells, which are easy to produce, stable in storage, and capable of surface binding, provide an effective platform for activating tumor-specific immune responses. Nevertheless, the safety issues should be carefully evaluated when applying tumor cell-based drug delivery systems.

Polyinosinic–polycytidylic acid (Poly I:C), a synthetic double-stranded RNA mimic, can boost immune responses and induce apoptosis of tumor cells [114]. Macrophages can deliver drugs to the tumors by recognizing tumor-secreted chemokines and cytokines. However, macrophage vectors may be reprogrammed to the M2 type in TIME. To amplify the antitumor activity of macrophages, poly(lactic-co-glycolic acid) (PLGA) nanoparticles encapsulating poly I:C were coupled onto the macrophage surface via a click chemistry reaction [115]. The biomimetic system delivering poly I:C suppressed primary and metastatic tumors by directly triggering apoptosis of tumor cells, promoting DCs maturation, and polarizing macrophage vectors to the tumoricidal M1 type. The in situ activation strategy provides a new insight for a macrophage-based drug delivery system to enhance antitumor immunity.

#### 2.3.2. Cell Membrane-Coated Nanoparticles

Cell membranes have been widely used to improve the biological properties of synthetic nanoparticles. By wrapping the nanoparticle core with the cell plasma membrane through extrusion, sonication, or electroporation, the obtained cell membrane-coated nanoparticles display the same surface markers with natural cells and copy natural cellular behaviors [116]. Different sources of cell membranes confer different properties to the encapsulated nanoparticles. For example, erythrocyte membranes can prolong the half-life of the nanoparticles and evade clearance by the immune system [117]; inflammatory cells can penetrate blood vessels and target tumors or inflammatory sites [118]; platelet membranes can target circulating tumor cells [119]; and immune cells, tumor cells, and stem cells have the potential to target tumor sites via homotypic recognition [120]. Hybrid membranes derived from diverse cell types can also be fabricated to exhibit multifunctionality [121]. Moreover, cell membranes can be modified through gene editing and surface engineering to enhance their target recognition and improve in vivo behaviors [122]. 

siRNA regulates gene expression through RNA interference (RNAi): cleaving mRNA after binding to an RNA-induced silencing complex (RISC) [11]. siRNA broadens the strategy for modulating therapeutic targets lacking drugs. In order to improve siRNA stability and reduce “on-target, off-tumor” toxicity, the tumor cell membrane was utilized to coat PLGA nanoparticles containing programmed cell death 1 ligand 1 (PD-L1) siRNA. Compared to bare PLGA nanoparticles, cell membrane-camouflaged nanoparticles were more easily taken up by tumor cells, resulting in a more effective PD-L1 knockdown [123]. Similarly, the mesenchymal stem cell membrane with tumor tropism was utilized for coating the PD-L1 siRNA and doxorubicin co-loaded polydopamine nanoparticle [124]. The drugs were targeted and delivered to the tumor site, where doxorubicin exerted antitumor cytotoxicity and upregulated the expression of PD-L1 to provide more targets for PD-L1 siRNA. Consequently, the combination of chemotherapy and gene therapy inhibited prostate cancer bone metastases without causing noticeable side effects. A biomimetic siRNA delivery system was also constructed by encapsulating a spermine-based nanoparticle compressing PD-L1 siRNA and a photosensitizer indocyanine green (ICG) with the macrophage cell membrane [125]. After specific uptake by tumor cells due to the recognition by Seglec-15 on a macrophage cell membrane, PD-L1 siRNA achieved a lysosomal escape for degrading PD-L1 mRNA, while IGG mediated photodynamic therapy for generating an in situ tumor vaccine. After the treatment, the intratumoral CD4^+^ and CD8^+^ T cells were upregulated by 8.2 and 9.5 times, respectively, and regulatory T cells (Treg) were downregulated. The nanoplatform with macrophage membrane camouflage improved the in vivo behavior of the loaded drugs, providing a viable strategy for immunotherapy based on siRNA. A hybrid membrane derived from macrophages and tumor cells with dual properties could also improve the delivery and lysosomal escape of siRNA with immune regulation capacity [126]. In addition, efficient delivery of siRNA could also be achieved by modifying the cell membrane surface with materials with targeting functions such as aptamers [127]. 

Tumor cell membranes as a source of tumor-associated antigens can be combined with immune adjuvants to build integrated platforms as vaccines. Kroll et al. used the melanoma cell membrane loaded with the CpG-containing PLGA nanoparticle to promote the uptake of CpG by DCs [128]. CpG significantly upregulated the levels of co-stimulatory molecules and promoted the secretion of proinflammatory molecules from DCs, enhancing the specific immune response to tumor antigens. The treatment with the integrated platform prolonged the median survival time of tumor-bearing mice from 20 days to 40 days, facilitating the development of personalized antitumor vaccines.

Cell membranes can also be genetically edited to acquire additional functions and thus be more suitable as drug delivery vehicles. For instance, genetically engineered cell membranes expressing influenza virus fusion protein hemagglutinin could promote lysosomal escape of encapsulated mRNA, providing a platform for vaccination and gene therapy [129]. The intricate composition of cell membrane components provides versatility, but also presents safety challenges. Therefore, bioinspired drug delivery systems constructed from synthetic biomaterials are more controllable. Phospholipid and glycolipid derivatives encapsulating mRNA-expressing T cell co-stimulatory receptors mimicked cell membrane-encapsulated nanoparticles for cell-specific delivery of mRNA in vivo, providing a reference for the bottom-up design of cell membrane-based biomimetic drug delivery systems [130].

#### 2.3.3. Extracellular Vesicles

Extracellular vesicles with a membrane structure released by cells are mainly divided into exosomes, microvesicles, and apoptotic bodies according to their origins and sizes [131]. The components in the extracellular vesicles, including lipids, glycoproteins, and nucleic acids inherited from the origin cells, are stabilized under the protection of the phospholipid bilayer. The lipid layer also allows extracellular vesicles to penetrate natural barriers in vivo to deliver the contents to target cells and participate in intercellular signaling [132]. Strategies for nucleic acid drugs loaded into extracellular vesicles can be categorized into endogenous and exogenous loading [133]. Endogenous loading is achieved by treating origin cells to pack the cargos into the vesicles, whereas exogenous loading is the introduction of drugs into vesicles by electroporation or loading reagents. Generally, exogenous loading such as electroporation is suitable for almost all nucleic acid drugs such as siRNA, mRNA, and CRISPR/Cas systems.

Loading of siRNA into extracellular vesicles is usually performed through exogenous strategies such as lipid anchoring, electroporation, and nanoparticle co-extrusion [134,135]. Modification of extracellular vesicles using biomaterials can further improve their targeting effects and cellular uptake. For example, to overcome the blood–brain barrier and TIME in glioblastoma, cerebrovascular endothelial cell-targeting peptides were coupled onto neuronal cell-derived extracellular vesicles to form delivery vehicles for PD-L1 siRNA [136]. The vesicles were engulfed by macrophages after administration, which could migrate to the irradiated inflammatory site after radiotherapy. Radiotherapy also upregulated the intratumoral PD-L1 level, providing targets for siRNA. An ApoA1-modified tumor-derived exosome coupled with siRNA targeting neutral sphingomyelinase type 2 also effectively targeted tumor cells and enhanced the antitumor activity of CD8^+^ T cells by reducing the level of PD-L1 [137]. A virus-derived fusogenic protein with pH sensitivity was introduced on extracellular vesicles obtained from M1-type macrophages [138]. PD-L1 siRNA was then loaded into vesicles by electroporation. In the acidic tumor microenvironment, the fusogenic protein mediated the membrane fusion of the vesicles and cell membranes, bypassing the endocytosis pathway to inhibit siRNA degradation. PD-L1 siRNA downregulated the expression of PD-L1 in tumor cells, and the proinflammatory cytokines in the M1 macrophage-derived vesicles reprogramed M2-type TAMs. Synergistic activation of antitumor intrinsic and adaptive immunity inhibited in vivo tumor growth significantly, indicating that extracellular vesicles from immune cells could act as an effective drug delivery system with tumor tropism and endogenous active components. In addition, tumor-derived exosomes enriched with tumor-associated antigens could also be developed as a vaccine with the delivery of nucleic acid adjuvants [139].

Cell-secreted vesicles naturally contain certain mRNA that can be directly utilized for disease treatment [140]. mRNA can be endogenously loaded into extracellular vesicles. By fusing two vesicles formed by transfecting a plasmid expressing a targeting antibody and a plasmid expressing a therapeutic mRNA into cells with the assistance of PEI, respectively, hybrid vesicles could be generated for specific mRNA delivery [141]. To achieve targeting of specific cells, CD64 was expressed on extracellular vesicles to bind antibodies (anti-CD71 and anti-PD-L1) that can target a glioblastoma tumor [142]. Engineered vesicles actively loaded with IFN-γ mRNA were generated in a high-throughput manner using nanosecond pulse electroporation. The nanovesicles would be internalized by tumor cells when binding to certain receptors and release mRNA. The secreted IFN-γ upregulated tumor major histocompatibility complex (MHC) class I expression, enabling the immune system to recognize tumor cells and thus reversing TIME. The adaptive vesicles have the potential to achieve efficient mRNA delivery by altering the coupled antibodies based on the protein overexpressed at the target site. While mRNA delivery systems utilizing extracellular vesicles for antitumor immunotherapy have not been extensively researched, certain technological advancements have provided references for their study, which include employing cellular nanoporation for large-scale production of mRNA-containing vesicles [143], enhancing mRNA encapsulation and delivery through stabilization with retrovirus-like capsids [144], and modifying vesicles with immune cell-targeting proteins to enhance uptake [145].

Extracellular vesicles can also be used to deliver CRISPR/Cas systems for effective in vivo gene editing. In vitro loading of CRISPR/Cas may cause changes in vesicle properties or introduce artificial components. Stranford et al. developed a strategy to encapsulate both cargo proteins (Cas9) and targeting proteins (CD2 single-chain fragment, CD2 scFv) in vesicles by abscisic acid-inducible dimerization [146]. The bioengineered vesicles effectively delivered gene editing system to CD2^+^ T cells, providing a versatile platform for targeted delivery of biologics to other cell types. To achieve in vivo gene engineering of T cells, extracellular vesicles containing CRISPR/Cas9 systems were decorated with antibodies for T cell-targeting and a mutant type of the vesicular stomatitis virus glycoprotein for cell fusion [147]. By using multiple targeting molecules toward human T cells, Cas9-packaging enveloped delivery vehicles were able to generate gene-edited CAR-T cells in humanized mice, establishing a programmable delivery mode for broadening therapeutic applications.

A concise overview of representative cell-derived nucleic acid delivery systems for antitumor immunotherapy introduced in this section is summarized in Table 1.

## 3. Conclusions and Perspectives

Nucleic acid drugs hold potential in cancer immunotherapy with plentiful targets and high specificity. CRISPR/Cas systems regulating immune cell functions [148], DNA encoding proinflammatory cytokines [149], mRNA expressing tumor antigens [18], siRNA knocking down immune checkpoints [150], and oligonucleotides directly activating immune cells [151] are promising strategies for stimulating antitumor immunity. Nevertheless, the progress of nucleic acid drugs is impeded since they are susceptible to degradation by nucleases and face challenges in cellular uptake due to their high molecular weight. Hence, the development of delivery systems has emerged as a pivotal aspect of nucleic acid drug discovery and advancement. Optimal delivery systems typically exhibit the following attributes: (i) good biocompatibility, biodegradability, and low immunogenicity; (ii) shielding nucleic acid drugs from serum nuclease degradation during the circulation; (iii) evading rapid clearance by the liver or kidneys; and iv) precise delivery of the drugs into target cells while sparing normal tissues from impact [152]. Several lipid nanoparticles for siRNA and mRNA delivery have been approved by the FDA for treating virus infections and genetic diseases, and a series of lipid nanoparticle-based mRNA tumor vaccines are under clinical evaluation [31,153,154,155]. Although lipid nanoparticles have assisted certain nucleic acid drugs to successfully enter the clinic, they still face challenges in achieving significant clinical benefits, such as difficulty in organ/cell-selective delivery and immunogenicity of lipid nanoparticles, and latent toxicity caused by lipid accumulation in tissues [156]. Therefore, there remains a crucial necessity to pioneer novel nucleic acid drug delivery systems to mitigate issues related to toxicity caused by artificial materials and off-target effects.

Biomimetic delivery systems derived from viruses/bacteria/cells have the potential to augment the therapeutic efficacy of nucleic acid drugs, particularly in the realm of immunotherapy, for the following several reasons: (i) precisely delivering cargos to target sites with the natural tissue tropism; (ii) enhancing the drug uptake by specific cells mediated by the inherent ligands; (iii) protecting the cargos from degradation and clearance; and (iv) modulating TIME with the endogenous activities. Additionally, biomimetic vectors can undergo further modifications through biological, chemical, and physical methods to enhance their effectiveness, specificity, controllability, and safety.

Biomimetic systems derived from various sources possess unique properties. Viruses are suitable for delivering DNA fragments encoding immunostimulatory molecules due to their high transduction efficiency. Oncolytic viruses, including H101 and T-Vec, have entered clinical practice for almost 20 years [157]. Toxicity has been a key barrier to viral therapy. Recently, strategies based on synthetic biology, including limiting viral replication in vivo or introducing tumor-specific response elements, could improve the safety of viruses [70,158]. For example, SynOV1.1, the first synthetic biology-engineered virus to enter clinical trials, is highly restricted to play a lethal role inside tumor cells and secrete pro-inflammatory cytokines [159]. The use of protein packaging or compression of nucleic acids to form virus-like particles is more biocompatible, but its transfection efficiency and encapsulation rate need to be improved. With the mining and modification of endogenous proteins, new protein vectors with high binding and transduction efficiency for nucleic acid delivery are expected to be developed.

Bacteria-derived vectors with tumor-targeting and immune-activating capabilities have the potential to carry a variety of nucleic acid cargos and be used in antitumor combination therapy. Engineered *Salmonella* secreting IL-12 and *Listeria monocytogenes* encoding tumor antigens have successfully activated the immune systems and yielded certain clinical benefits [160]. However, Grade 3 or higher immune-related side effects often occur in the clinical trials of antitumor bacterial therapies. Balancing the safety and efficacy of therapeutic bacteria is challenging because removing toxic components may compromise their effectiveness. Selecting appropriate patients or using synthetic biology to enable bacteria to express toxic factors only in specific environments holds promise for addressing this issue.

Cell-derived platforms with multifaceted endogenous functionality and low immunogenicity have the potential to accommodate both safe and effective delivery of nucleic acid drugs. Nevertheless, cell-based nucleic acid drug delivery systems remain largely in the preclinical stage or demonstrate limited efficacy in clinical trials. For example, whole-tumor cell vaccines engineered to secrete GM-CSF (GVAX) have not yielded meaningful outcomes in patients bearing solid tumors and hematologic malignancies [161]. The complicated in vivo behavior of the cells, the high heterogeneity of cell membranes, the uncontrollable drug loading process of extracellular vesicles, and the compensatory effect of the tumor immunosuppressive microenvironment limit the large-scale production and clinical translation of cell-derived nucleic acid delivery systems.

Biomimetic nucleic acid delivery systems face many challenges in their development and clinical translation, and more efforts are required. First, nucleic acid drugs need to be carefully designed to enhance efficiency or reduce off-target toxicity. Intensive studies of tumor-immune interactions are expected to provide new targets and design strategies for developing new nucleic acid drugs. Second, the pharmacokinetics and pharmacological mechanisms of biomimetic systems differ from those of traditional drugs and conventional drug formulations, making it challenging to predict their in vivo behaviors. Studying the biomimetic systems in humanized animal models may help predict their behavior in the human body, and new evaluation methodologies need developing. Third, the complex tumor immune microenvironment and patient heterogeneity lead to variable treatment outcomes, highlighting the need for combined and personalized therapeutic strategies. Last but not least, the stability and standardized production of biotherapeutics are challenging due to their specific requirements, such as the inability to use high-temperature sterilization to remove unwanted pathogens, as it may affect the drug’s activity. 

## Figures and Tables

**Table 1:** Cell-derived nucleic acid delivery systems for activating antitumor immune response.

Nucleic Acid Drug	Delivery Platform	Strategy of Constructing the Biomimetic Nucleic Acid Drug Delivery System	Mechanism of Activating Antitumor Immune Response	Antitumor Effects	Ref.
CpG	Cryo-silicified tumor cells	Polyethyleneimine (PEI)-adsorbed cryo-silicified tumor cells were coated with CpG.	CpG activated Toll like receptor (TLR) 9 and silicified tumor cells served as the vaccine.	The vaccine eradicated the tumors in C57BL/6 mice bearing ovarian cancer.	[113]
Poly I:C	Macrophages	Poly(lactic-co-glycolic acid) (PLGA) nanoparticles encapsulating poly I:C were coupled onto the macrophage surface.	Poly I:C triggered apoptosis of tumor cells, promoted dendritic cells (DCs) maturation, and polarized macrophage vectors to the M1 type.	The biomimetic system induced 84.9% tumor cell apoptosis in vivo and inhibited lung metastases by 74.9% in 4T1 tumor-bearing mice.	[115]
Programmed death ligand 1 (PD-L1) small interfering RNA (siRNA)	Tumor cell membrane	Tumor cell membrane was utilized to coat PLGA nanoparticles containing PD-L1 siRNA.	Cell membrane-coated nanoparticles were more easily taken up by tumor cells, resulting in a more effective PD-L1 knockdown.	The nanoparticle induced cytotoxicity in the source cells of the cell membrane coating.	[123]
PD-L1 siRNA	Mesenchymal stem cell membrane	Mesenchymal stem cell membrane was utilized for coating PD-L1 siRNA and doxorubicin co-loaded polydopamine nanoparticle.	Doxorubicin exerted antitumor cytotoxicity and upregulated the expression of PD-L1, which could be inhibited by PD-L1 siRNA.	The biomimetic nanoparticle inhibited PC-3 cell growth both in vitro and in vivo.	[124]
PD-L1 siRNA	Macrophage cell membrane	Spermine-based nanoparticle compressing PD-L1 siRNA and a photosensitizer ICG was encapsulated with a macrophage cell membrane.	PD-L1 siRNA degraded PD-L1 mRNA, while IGG mediated photodynamic therapy for generating the in situ tumor vaccine.	The combination therapy achieved the apoptotic rate of 46.1% in vitro and the tumor growth inhibition rate of ~80% in vivo.	[125]
siRNA targeting fibrinogen-like protein 1	Tumor cell–macrophage hybrid membrane	PLGA nanoparticles encapsulating siRNA and metformin were coated with the hybrid membrane.	Metformin and siRNA synergistically promoted T-cell-mediated immune responses.	The nanoparticle achieved an apoptosis rate of 75.71% in 4T1 cells in vitro and a tumor inhibitory rate of 97.3% in vivo.	[126]
CpG	Melanoma cell membrane	Melanoma cell membrane was loaded with a CpG-containing PLGA nanoparticle.	CpG induced maturation of DCs and the tumor cell membrane provided tumor antigens.	The vaccine prevented tumor occurrence in 86% of the mice and inhibited tumor growth.	[128]
PD-L1 siRNA	Neuronal cell-derived extracellular vesicles	Cerebrovascular endothelial cell-targeting peptides were coupled onto neuronal cell-derived extracellular vesicles to form delivery vehicles for PD-L1 siRNA.	Radiotherapy upregulated the intratumoral PD-L1 level, providing targets for siRNA.	The combination therapy inhibited glioblastoma in vivo and extended the median survival time from 22.5 to 47 d.	[136]
siRNA targeting neutral sphingomyelinase type 2	ApoA1-modified tumor-derived exosome	ApoA1-modified tumor-derived exosome was coupled with cholesterol-decorated PD-L1 siRNA.	The downregulation of neutral sphingomyelinase type 2 reduced the level of PD-L1.	The exosome achieved the PD-L1 silencing efficiency of 94.07% at the protein level and significantly delayed HepG2 tumor growth.	[137]
PD-L1 siRNA	M1-type macrophage extracellular vesicles	PD-L1 siRNA was loaded by electroporation into vesicles expressing a virus-derived fusogenic protein with pH sensitivity.	PD-L1 siRNA downregulated the expression of PD-L1 in tumor cells, and the proinflammatory cytokines in the M1 macrophage-derived vesicles reprogramed M2-type tumor associated macrophages (TAMs).	The combination immunotherapy achieved the tumor inhibition rate of over 80% in vivo.	[138]
Interferon (IFN)-γ mRNA	Extracellular vesicles expressing CD64	Antibodies-decorated vesicles loaded with IFN-γ mRNA were generated with nanosecond pulse electroporation.	The secreted IFN-γ upregulated tumor major histocompatibility complex (MHC) class I expression, enabling the immune system to recognize tumor cells.	The vesicle prolonged the median survival time from 29 to 53 d in GL261-bearing mice.	[142]
sgRNA and Cas protein	Extracellular vesicles expressing cell-targeting antibodies	Extracellular vesicles containing CRISPR/Cas9 systems were decorated with antibodies for T cell targeting and a mutant form of the vesicular stomatitis virus glycoprotein for cell fusion.	The engineered vesicles generated gene-edited chimeric antigen receptor (CAR)-T cells for killing tumor cells.	The in vivo-generated CAR-T cells depleted CD19 B cells.	[147]
